# Children in the Anthroposophical Education System Have Lower Rates of Obesity, and Higher Rates of Health Promoting Behaviors

**DOI:** 10.3390/nu15143088

**Published:** 2023-07-10

**Authors:** Moran Blaychfeld Magnazi, Anat Gesser-Edelsburg, Yafit Itzhaky, Ronit Endevelt, Naomi Fliss Isakov

**Affiliations:** 1 Nutrition Division, Public Health Services, Ministry of Health, Jerusalem 9101002, Israel; ronit.endevelt@moh.gov.il (R.E.); naomi.fliss@moh.gov.il (N.F.I.); 2 School of Public Health, the Health and Risk Communication Laboratory, University of Haifa, Haifa 3498838, Israel; 3 Department of Public Health Nursing, Public Health Services, Ministry of Health, Jerusalem 9101002, Israel; 4 School of Public Health, Faculty of Medicine, Tel Aviv University, Tel Aviv 6997801, Israel

**Keywords:** dietary norms, anthroposophical education, childhood obesity, primary prevention

## Abstract

Background: The anthroposophical philosophy is a holistic educational and lifestyle approach. Limited information exists on the health-promoting behavioral norms and obesity rates among children living anthroposophical vs. conventional lifestyles. Aims: This study aims to compare the prevalence of childhood obesity, and parents’ perceptions of their children’s food environment, between anthroposophical and conventional education systems. Methods: We performed a cross-sectional analysis of the National Anthropometric Measurement Survey for first grade students in Israel, comparing anthroposophical schools with matched conventional schools. Additionally, an online survey was distributed among parents of children in both school systems, assessing children’s eating norms and dietary intake. Results: Overweight and obesity rates were higher among students in conventional schools (*n* = 205,500) compared to anthroposophical schools (*n* = 2247) (11.2% vs. 9.6%, and 7.8% vs. 4.8%, respectively; Pv < 0.001). Anthroposophical schools were perceived by more parents to have health-promoting curricula, health promoting teacher behavior, and health promoting social dietary norms, while their children’s dietary intake was perceived as healthier both in school and in the after-school, social, and familial environment (Pv < 0.001). Conclusions: Children in anthroposophical education exhibited lower overweight and obesity rates, and engaged in more health-promoting behaviors. Further research is needed to explore the relationship between the anthroposophical lifestyle and childhood obesity, and to identify effective anthroposophical strategies for health promotion among children.

## 1. Introduction

Childhood obesity is a major public health concern worldwide. The World Health Organization (WHO) has recognized childhood obesity as one of the most significant challenges of the 21st century [1]. Childhood obesity is likely to persist into adulthood, therefore, current trends predict a population of adults exposed to the impact of excess adiposity for a long period of time. This, in turn, is likely to lead to more serious health consequences at both the individual and the population level [2,3]. Obesity is a complex multifactorial disease defined by excessive adiposity, and is linked to an increased risk for many non-communicable diseases (NCDs) [4], including cardiovascular diseases (CVDs), cancer, type 2 diabetes mellitus (T2DM), and chronic respiratory diseases, including obstructive sleep apnea (OSA) [5,6,7].

Overweight and obesity affects almost 60% of adults, and one in three children (29% of boys and 27% of girls aged 7–9 years) are living with overweight or obesity in the WHO European Region [8,9]. In Israel, as part of student health services, starting in 2013, growth assessment has routinely been performed for students in the first grade. According to the data gathered, the prevalence of overweight and obesity among male first grade pupils in Israel from 2018 to 2019 and 2020 increased from 17.7% to 18.3% and 19.7%, respectively. Among girls, the prevalence of overweight and obesity was 18.9%, 19.2%, and 20.7%, respectively.

Childhood obesity is multifactorial, ref. [10] encompassing additive and multiplicative interactions between genes and environment that may be reflected in one’s food consumption, a sedentary lifestyle, and sociocultural provocations [11].

The early-life risk factors of childhood obesity have been described, and include sociodemographic characteristics (such as a low socioeconomic status and low health literacy), behavioral factors (such as not being breastfed as an infant, exposure to unhealthy complementary foods in early life, childhood consumption of an unhealthy diet, a sedentary lifestyle due to early and long exposure to screens, and a low level of physical activity), and an unhealthy food environment, including an abundance of cheap unhealthy foods in local markets, exposure to extensive food marketing, a poor quality of food served in the educational system, etc. [12,13].

The food environment within the educational system holds significant potential for influencing the diet of children and adolescents by several means. First, a school-served lunch contributes a substantial proportion of children’s total daily calories (19–50% of calories) [14]. Second, the education system may choose to emphasize the healthy diet and lifestyle in their curriculum. Similarly to schools, other educational or recreational facilities for children have been shown to play an important role in shaping children’s dietary choices [15].

Among the different alternative educational systems, “Waldorf education” or, alternately, “anthroposophical education” aims to educate holistically healthy children, through a formula that rejects the conventional distinction between education and health, and also focuses on “protecting childhood” from the perceived dangers of modern society [16,17]. It includes references to mental health, the maintenance of a healthy diet, and the performance of physical activity [18]. The nutritional agenda in anthroposophical education is guided by the belief that food plays a crucial role in the physical, mental, and spiritual development of a child. The anthroposophical education system advocates in favor of a vegetarian diet, and all kindergarten served meals are vegetarian. The system also often places a strong emphasis on prioritizing fresh, whole foods, home-cooked meals, and garden-grown fruit and vegetables. On the other hand, it encourages pupils to avoid processed foods and artificial ingredients. There is also a strong emphasis on the interconnectedness of food, agriculture, and the environment. These concepts are expressed through routine activities which are incorporated into the curriculum, such as gardening, communal cooking, and recycling [19,20]. Parents who choose to educate their children in an anthroposophical school may incorporate these concepts further into their home and social environment. Taken together, these school and familial behavioral norms may play a crucial part in the prevention of childhood obesity. Moreover, students of anthroposophical education have been found to have better health indicators, such as fewer allergies and stomachaches, and lower rates of hypertension, eczema, and antibiotic use, compared to students in conventional education [21]. Former pupils of Waldorf schools were also found to better endure significant emotional loads, life stress, and crises [18]. Moreover, mothers to children in anthroposophical schools have been shown to have had a lower BMI before and after their pregnancy, compared to mothers of children in conventional schools [22]. This is assumed to reflect a holistic lifestyle, which includes a plant-based, minimally processed diet, and an active lifestyle, which are fundamental components of the anthroposophical lifestyle philosophy. Parents’ choice to enroll their children in an anthroposophical education system is assumed to reflect other aspects of their behavior. In turn, these holistic lifestyle characteristics are postulated to promote lower pediatric obesity rates.

In Israel, the anthroposophical education system includes over 25 elementary schools. To date, a comprehensive study examining familial and community lifestyle characteristics, parental attitudes regarding their children’s food environment, and anthropometric measurements between anthroposophical and conventional educated students has not yet been performed.

We therefore aimed to: (1) assess the difference in the prevalence of childhood overweight and obesity among first grade students between the anthroposophical and conventional educational systems in Israel, and (2) identify the differences in parental attitudes regarding their children’s food environment between the anthroposophical and conventional educational systems.

## 2. Materials and Methods

We conducted two cross-sectional phases.

Phase 1: Assess the prevalence of childhood overweight and obesity among first grade students between the anthroposophical and conventional educational systems.

A cross-sectional study was conducted, based on data gathered from the National Anthropometric Measurement Survey of the Ministry of Health.

### 2.1. Study Population

Children in the first grade who had undergone screening and anthropometric measurements as part of the National Anthropometric Measurement Survey of the Ministry of Health between 2018 and 2020. All children in anthroposophical schools were included in the study. In Israel, anthroposophical schools are regional, so, for each anthroposophical school, we individually matched all conventional-education schools from the same city or region, in an attempt to minimize the selection bias, and control for municipally associated socio-demographic characteristics. 

### 2.2. Data Collection

The National Anthropometric Measurement Survey of the Ministry of Health is an extensive screening program conducted annually, covering 90% of the children enrolled in the first grade in Israel (age 6–7 years) [23]. It is a national initiative to monitor childhood obesity rates, for the promotion of prevention and treatment practices. Measurements are performed by dedicated and trained nurses according to a systematic protocol, and include weight (kg) and height (m) measurement, body mass index (BMI) calculation (BMI = weight/height^2^), and BMI percentile categorization, according to the World Health Organization (WHO) BMI growth charts. The anthropometric categorization of each student was made as: severe obesity: BMI > 99th percentile, obesity: 97th < BMI ≤ 99th percentile, overweight: 97th ≤ BMI < 85th percentile, normal weight 3rd < BMI ≤ 85th percentile, and underweight: BMI ≤ 3rd percentile. The sum of the students who were candidates for anthropometric evaluation, the sum of the students eventually measured, and the sum of the students in each anthropometric category were recorded. We excluded measurements from classes with an irregular number of children (>45 or <10 children per class), and in which a large proportion of students were not measured (>80% of candidates). The total number of measured students, and the number of students categorized to each of the anthropometric categories were summed, from anthroposophical and conventional schools separately. The proportion of students in each anthropometric category was calculated separately for anthroposophical and conventional schools in Israel. From each of the schools included, measurements from three consecutive school years were included.

Phase 2: Identify parental attitudes regarding their children’s food environment, between the parents of children in anthroposophical and conventional educational systems. A cross-sectional study was performed via an anonymous online survey filled in by parents of elementary school children. Participation was voluntary; no incentives were offered.

### 2.3. Study Population

A convenience sample of parents of children in elementary school, reached using a snowball sampling method. The survey was constructed using Qualtrics Survey Software (https://www.qualtrics.com/, accessed on 14 June 2023), and distributed between March and May 2021 through the following social media platforms: (1) WhatsApp groups of parents from anthroposophical and conventional schools, (2) Facebook groups of parents from different regions in Israel, and (3) on the researchers’ private Facebook accounts. Survey participants were encouraged to distribute the questionnaire link to other parents among their friends and family, in order to achieve maximum exposure. From all the responses to the questionnaire (parents who opened the questionnaire link), we included only complete questionnaires (with a response to >90% of questions), from parents with at least one child studying in elementary school (first–sixth grade) in either anthroposophical or conventional education. Responses were excluded if they were from parents of children in the ultra-Orthodox educational system, because this system does not include anthroposophical schools.

### 2.4. Research Tools

The study questionnaire was developed and validated using conventional methods [24]. The questionnaire was developed by a multidisciplinary expert committee. Its aim was to elucidate the perceived difference between anthroposophical and conventional schools, in aspects related to children’s food environment and dietary intake. The study questionnaire structure and content validation were assessed using a pilot sample of 15 participants. The participants were asked to provide feedback on the readability and understandability of the questionnaire, and the time needed to fill out the questionnaire. Changes were made to the questionnaire accordingly [24]. The questionnaire includes questions regarding the perceived role of the educational system in imparting a healthy lifestyle, actual health-promoting behaviors at school and in kindergarten, and the nutritional norm at after-school activities, at community events, and at home. Questions further referred to parents’ perceived frequency of food consumption by their children, parents’ body image, and personal and familial demographic parameters. Lastly, the questionnaire included open questions, by which the parents were given the opportunity to provide free-text answers regarding the dietary habits and norms in their children’s educational system. All, but the open questions, were multiple choice, and answers were coded to qualitatively reflect a parent’s identification with an educational measure. The full version of the questionnaire is depicted in Appendix A.

After the questionnaire was constructed, and in accordance with the answers from the pilot group, Cronbach’s alpha value was calculated for statements that appeared to be associated with eight measures of theoretical significance, in order to validate eight index scores. All statements which were highly correlated with a measure were scored and the mean of all scores under the same measure was calculated as the index score (Table 1). For instance, all statements about parents’ perceived role of the educational system in imparting a healthy lifestyle were scored (questions 12.1-4), and the mean score of these statements was presented as the total index score. Higher index scores represent a stronger identification with the measure in question.

The frequency of food consumption was classified according to a scale of 0–3; a high-frequency consumption of healthy foods was classified as 3, and a high frequency of unhealthy foods was classified as 0.

### 2.5. Statistical Analysis

All statistical analyses were performed using the SPSS software, version 25. Continuous variables are presented as means± standard deviation (SD), and nominal variables as proportions. The level of significance was set at 5%.

For the first phase: The difference in proportions of each anthropometric category between the conventional and anthroposophical students was evaluated using the chi-square test. For the second phase: The background data of parents was displayed using descriptive statistics: the average and standard deviation for continuous variables, and percentages for categorical variables. Parent’s age distributed normally, and was compared between parents to children in anthroposophical and conventional schools using an independent sample *T*-test. The family number of children did not distribute normally, and was compared between the groups using the Mann–Whitney test. Categorical variables were compared between the groups using the chi-square test. The content analysis approach was used for the data analysis of the open questions [25]. The narratives were analyzed, to determine the key themes. The analysis involved indexing the data, sorting and selecting quotes in order to place them in the appropriate thematic category, and developing a final interpretation.

## 3. Results

### 3.1. Phase 1: Prevalence of Childhood Overweight and Obesity among First Grade Students between Anthroposophical and Conventional Educational Systems

Altogether, throughout 2018–2020, we collected data from 205,500 students from conventional schools, and 2247 students from anthroposophical schools (representing 96.8% and 92.4% of students in the schools which were included in the analysis). The schools included in the analysis were from eleven regions of Israel (Jerusalem *n* = 84,518, 40.7% of total study population; Tel Aviv *n* = 59,766, 28.8%; Petah Tikva *n* = 23,164, 11.1%; Ramla *n* = 15,655, 7.54%; Rehovot *n* = 13,945, 6.7%; Haifa n = 6864, 3.3%; North *n* = 3838, 1.8%). The highest proportion of students from anthroposophical schools were from the Tel Aviv region (25.2% of students), followed by Jerusalem (19.0%), and the Northern district (16.8%).

Significant differences were found between the anthroposophical and conventional education systems in the proportion of all anthropometric categories. These differences were detected in both genders. Overweight and obesity rates were significantly higher among students in the conventional education system compared to those in the anthroposophical education system (11.2% and 7.8%, vs. 9.6% and 4.8%, respectively; Pv < 0.001 for both). On the other hand, the proportion of normal weight, and underweight, was lower in students in the anthroposophical education system, compared to in the conventional education system. The differences in the proportions of students in anthropometric categories between the anthroposophical and conventional educational system is presented in Table 2.

### 3.2. Phase 2: Attitudes and Perceptions of Parents Regarding Their Children’s Food Environment

The percentage of complete response to the survey was 63.8% (214/335 participants who opened the questionnaires). Parents of children in anthroposophical schools (*n* = 72), were compared to parents of children in conventional schools (*n* = 142).

Among parents of children in anthroposophical schools, 89% reported previous education in an anthroposophical kindergarten, and 62.5% reported having ≥5 years of experience in the anthroposophical education system.

The parent’s age, gender, and dietary pattern differed significantly between parents of children in anthroposophical and in conventional schools, though other demographic characteristics, including years of education, income level, number of children, and non-single parents, did not (Table 3).

### 3.3. Parental Perception of Their Child’s Dietary Intake and Food Environment, and Their Own Body Image

Parents of children in anthroposophical schools perceive the educational system’s role in maintaining the student’s healthy lifestyle significantly more meaningful, compared to the parents of children in the conventional system (Pv < 0.001). They perceive their children’s school curriculum, and teacher’s behavior, to be significantly more health-promoting, and their children’s extra-curricular nutritional behavior norm to be healthier, compared to parents of children in conventional schools. Parents of children in anthroposophical schools reported their children’s overall dietary habits to be healthier, including a higher intake of fruit, vegetables, and legumes, and a lower intake of snacks, compared to parents of children in conventional schools. Interestingly, they also reported a higher body image compared to parents to children in conventional schools (Table 4).

Among parents of children in anthroposophical schools, 33.3% agreed with a statement that the educational system can build the foundations for a healthy diet and lifestyle in childhood and adulthood, 40.3% stated that they believe the school teachers provide a healthy environment for their children in school, and 95.8% stated that the school provides instructions and recommendations for parents regarding food provided during afterschool activities (birthday parties, holidays, trips, etc.), compared to 9.1%, 3.5%, and 65.2%, respectively, among parents of children in conventional schools (Pv < 0.001 for all). Parents of children in anthroposophical schools stated that healthy diet principles are naturally integrated in their children’s everyday routine (88.9%, compared to 16.9% among parents of children in conventional schools, Pv < 0.001). Fifty-four percent of parents of children in anthroposophical schools stated that they perceive the dietary norms in school to be strongly coherent with that of their home, compared to 10.6% in conventional schools (Pv < 0.001). Moreover, a significantly lower proportion of parents of children from anthroposophical schools stated that they felt that other parents from their school supported their dietary choices, compared to parents of children from conventional schools (23.7% vs. 52.9%, *p* < 0.001).

Interestingly, both the parents of children in anthroposophical schools, and in conventional schools stated that the quality of their children’s diet is important to them (56.9% vs. 66.9%, respectively; Pv = 0.426), and that their children eat vegetables and fruit on a daily basis (95.8% vs. 90.1%, respectively, Pv = 0.144; and 87.5% vs. 80.3%, respectively, Pv = 0.413). The perceived difference in the children’s intake, between parents of children in anthroposophical and conventional schools, was in the proportion of daily intake of whole grains (66.7% vs. 37.6%, respectively, Pv < 0.001), legumes (40.3% vs. 8.5%, respectively, Pv < 0.001), and nuts and seeds (33.3% vs. 14.8%, respectively, Pv < 0.001). They also reportedlower weekly intake of savory snacks (0.0% vs. 2.8%, respectively, Pv < 0.001), sugar-sweetened beverages (1.4% vs. 10.6, respectively, Pv = 0.002), and eating while watching television at school (0.0% vs. 43.0%, respectively, Pv < 0.001). Finally, 61.1% of parents of children in anthroposophical schools stated that the quality of their children’s diet was a major contributor to their selection of a school system, compared to 7.7% among parents of children in conventional schools (Pv < 0.001).

Of the 214 participants, 200 parents answered the open questionnaire questions (65 parents of children in anthroposophical schools, and 135 parents of children in conventional education). When parents were asked about the positive aspects of the dietary norms at school, 12 (8.8%) parents of children in conventional schools reported that there were no good things at all to mention. On the other hand, when asked to elaborate on the negative aspects, 27 (41%) parents of children in anthroposophical school reported that there were no bad things at all to mention, vs. 33 parents (24%) of parents of children in conventional schools. The qualitative analysis, and the categorization into central themes, are presented in Table 5.

## 4. Discussion

The primary objective of this study was to compare anthropometric measurment between children in anthroposophical and conventional education, and to investigate the potential underlying reasons for the observed differences in the children’s weight status. 

In our analysis, overweight, obesity, and severe obesity rates in conventional schools in Israel were 19%. These rates are relatively low compared to European countries, as demonstrated by the recently published World Health Organization reports [26]. Israel is composed of a multiethnic population, with traditional Mediterranean diet heritage, but with an acceleration in sugar-sweetened beverages and ultra-processed foods intake during the last decade.

We used data from the National Anthropometric Measurement Survey of the Ministry of Health, performed annually, in a systematic manner, across all schools in Israel. Overweight and obesity rates were significantly more prevalent among children in conventional schools, compared to in anthroposophical schools. Obesity rates were 1.6 times higher among children in conventional schools, compared to those in anthroposophical schools. These were seen in boys, and even more so in girls. For this analysis, In Israel, anthroposophical kindergartens and schools are distributed across the entire country, and act as regional schools. Therefore, the matching of anthroposophical schools to all conventional schools per city/region aimed at reaching a wide population, and minimizing the selection bias. As the results of the National Anthropometric Measurement Survey of the Ministry of Health were analyzed anonymously, no sociodemographic characteristics of students were available for a comparison between the education systems. Moreover, whereas conventional schools may be classified according a national sociodemographic scale, according to the characteristics of residents in their close proximity, this classification is not relevant for regional anthroposophical schools. Advantages of this analysis include the large sample size, widely distributed across Israel, and standardized measurement methods. We were able to include in our analysis all anthroposophical schools in Israel, meaning that this population of students was unbiased, and could be assumed to be represented. On the other hand, the inability to account for the socioeconomic status of children between these comparison groups is a major limitation of this analysis. As anthroposophical schools in Israel are private, and conventional schools are public, a potential confounding by socio-economic status is possible [27], thus a selection bias may not be overruled, and this remains a probable confounder for the results. Also, a major limitation of this analysis is the cross-sectional design, which could not demonstrate a temporal association and a causal link between children’s educational system, their associated diet and lifestyle, and children’s anthropometric status.

Next, we conducted an anonymous semi-qualitative survey among parents of children in both school systems, to better understand their perceptions regarding their children’s food environment at school, at home, and at social events. Parents were asked to describe the perceived interaction and alignment between their children’s educational-system, home, and after-school dietary behaviors and norms. The results show that both groups of parents state that they rank the dietary habits of their children as important, but a higher proportion of the parents to children in anthroposophical schools stated that their children’s diet was a major contributor to their selection of a school system. They perceive the educational system as having a significant role in maintaining their children’s healthy lifestyle, and that healthy diet principles are naturally integrated in their children’s everyday school routine. These results are compatible with previous reports. In a systematic review that examined the association between school characteristics and pupils’ development of obesity, a healthy school food environment was significantly associated with lower rates of obesity or obesity trajectory. Other factors associated with lower obesity rates were a higher parental education and socio-economic status, meeting the recommended recess and physical education time, a suburban area compared to a rural area, and a higher parental involvement in school [28]. Interestingly, in our study, parents also described significant differences in children’s eating patterns further into the day, at after-school activities, and during social events, playdates, birthday parties, etc. These occasions may be a major contributor to the total intake of sweets and snacks among children, and may pave the way to their adulthood dietary norms [29,30]. Parents of children in anthroposophical schools also reported a higher sense of support from other parents in their school, regarding their preferred dietary practices, and the perception of body image was more positive among parents of children in anthroposophical education, compared to parents of children in conventional education. Although parents are thought to exert the primary influence on their children’s dietary and physical-activity behaviors, a study of weight trajectory modelling from the age of 2 to 7 years clearly showed that risk factors for an unhealthy body weight and poor diet extend beyond children’s homes [31]. Evidence suggests that children’s food environments, mainly in schools, childcare settings, and children’s recreational facilities, play an important role in supporting healthy food choices and limiting exposure to unhealthy foods and beverages [15]. It seems from our results that parents of children in anthroposophical schools perceive a stronger link between the dietary behavioral norms of children in school and after-school. The fact that both groups of parents reported a high importance and awareness of healthy diets and lifestyle reveals the difficulty that parents of children in conventional schools face in their attempt to maintain a continuous healthy lifestyle, against the dietary norms of their surroundings.

Finally, we also detected significant differences in the reported intake of children. Though both groups of parents reported daily intake of vegetables and fruit, parents of children in anthroposophical schools reported higher daily intake of whole-grains, legumes, and nuts and seeds, and lower intake of processed meat, savory snacks, and sugar-sweetened beverages. Importantly, parents from both groups did not differ in education level, family income, and family structure, implying that socioeconomic differences are not strong confounders of these results.

The main limitation of this analysis refers to the study questionnaire. Parents were not asked about a family history of obesity, or non-dietary lifestyle characteristics which may affect their children’s weight. We could not account for the history of breastfeeding [32], performing physical activity [33,34], screen time [35], etc., all of which are established risk factors for obesity. Furthermore, besides parental perceptions of their children’s dietary behaviors and norms, their child’s anthropometrics were not collected, so an association between these factors was not assessed. Additionally, chronic diseases, or emotional or developmental difficulties were not evaluated. These could be found in higher proportions within the anthroposophical education system, as it places a greater emphasis on personal special needs as part of its philosophy [36]. The anthroposophical education system in Israel is characterized by a higher teacher/pupil ratio and smaller classes, enabling a more personal relationship between teachers and students, and between peers. The fundamental difficulties of children which lead to the anthroposophical choice may reflect in eating developmental difficulties, which may explain the higher rates of underweight detected in this population. This hypothesis should be tested in a further studies. Last, a major limitation of this analysis is the cross-sectional design, which could not demonstrate a temporal association and a causal link between children’s educational system, their associated diet and lifestyle, and children’s anthropometric status.

The results of this study support previous studies determining the social and environmental risk factors for childhood obesity [11,12,13,14]. These influences are found in many aspects of a child’s life, including school [37], healthcare facilities [37,38], the neighborhood, and other community settings [39]. 

## 5. Conclusions

In conclusion, this study sheds light on a population of children in anthroposophical education who are characterized by a more preferable distribution of BMI, and potentially a healthier dietary environment, compared to children in conventional schools. According to the results, the anthroposophical lifestyle, as represented by dietary habits in the educational system and household, may represent a healthier environment, and a desirable model of promoting healthy-eating habits and normal weight maintenance. These findings substantiate the existing literature, and underscore the crucial role of a child’s environment in fostering healthy eating habits. Importantly our results show that the anthroposophical philosophy regarding diet, physical activity, and sustainability is not restricted to the educational system, but persists onward into children’s afterschool, social, and familial environments. By carefully studying and learning from these positive deviations, it is possible to identify and implement effective strategies to promote a healthy lifestyle in conventional education settings. Future studies should assess the association between various aspects of the anthroposophical lifestyle, and obesity prevention in larger populations and in prospective studies.

## Figures and Tables

**Table 1:** Conceptual aspects of children’s food environment, represented by eight food-environment indexes.

Index Name	The Questions	Alfa Cronbach	Index Scoring Range
Perceived role of the educational system in imparting a healthy lifestyle	* I believe that the educational system imparts nutritional habits that outline a way of life (Q12-1). I believe that teachers and care-givers in an educational setting should provide children with a healthy environment (Q12-2). In my opinion, the teachers of my child’s school promote a healthy dietary pattern, which they maintain themselves (Q12-3). In my opinion, the teachers of my child’s school care about his/her nutrition (Q12-4).	0.866	1–5
Perceived health-promoting behavior of teachers and school curriculum ^1^	< - On school special occasions (such as a trip, graduation party, teachers’ day, birthday celebrations, etc.), the food served usually includes snacks (Q15-1). On school special occasions, organizers try, as much as possible, to use reusable utensils (Q15-5). ** The teacher usually goes out with the class for a nature walk once a week (Q13-1). Most mornings, students preform physical activity, such as jumping rope/running as part of the curriculum (Q13-2). Usually, the teacher takes the class to the garden, to plant fruit or vegetables. When it is time to harvest them, the children can taste them (Q13-3). The school has strict guidelines regarding the nutritional content of food brought from home, and parents are instructed by the teacher about the permitted content of the lunch box/between meal snack, birthday parties, festivals, trips (Q13-4). If nutrition guidelines are given to students and parents, the teachers usually monitor the implementation of the guidelines (Q13-5). In my children’s curriculum, there are lessons dedicated to lifestyle, nutrition and health (Q13-6). At school the concept of a healthy diet is integrated naturally into different activities, such as food preparation, songs, vegetable gardens, arts and crafts (Q13-7). The teacher sits and eats breakfast with the children (Q13-8). Before breakfast, the teacher gathers the children and carries out a small ritual/says a prayer (Q13-9). < - Sometimes, during meals at school, children watch a program or movie (Q13-10).	0.877	1–0
Perceived after-school birthday celebrations.	** Children born in the same month usually celebrate birthday parties together (Q14-1). When class birthday parties are held in the afternoon, the parents usually make sure to prepare homemade food, such as casseroles/soup, sandwiches, salads/vegetables, pasta, etc. Later, there is also a birthday cake (Q14-2). < - When class birthday parties are held in the afternoon, snacks, sweets, vegetables, and sometimes pizza or sandwiches are served. Later, there is also a birthday cake (Q14-3).	0.817	0-1
Perceived after-school playdate-hosting norms	* Most parents who host kids for afternoon playdates offer fruit and vegetables (Q15-2). When my child is hosted for a playdate, snacks and sweets are often offered (Q15-3).	0.738	1–5
Perceived health-promoting behaviors in kindergarten	* In preschool, children are encouraged to prepare different foods by themselves, such as cutting up vegetables/soup/picking olives/pastry/jam and others (Q16-1). At preschool, the children participate in all processes of food preparation including cleaning up (Q16-2). The food my child ate during his time at preschool was very healthy (Q16-3). My child’s teachers usually promote healthy nutrition, and they themselves have healthy dietary habits (Q16-4). My child’s teachers care about his/her nutrition (Q16-5).	0.900	1–5
Perceived difference between the accepted eating pattern at home and that of friends and family members	* When my child is on a playdate at a friend’s or family member’s home, where the food served is different compared to that in my home, I feel uncomfortable (Q23-1). My child enjoys eating at other people’s homes when he is exposed to foods less heathy than the food served at home (Q23-2). I think my child feels conflicted when he meets with friends or family, where the food served is different to that served at home (Q23-3).	0.727	1–5
Perceived parents’ body image	For me, it is important to be very slim, in accordance with the beauty ideal (Q25-1). In order to achieve the beauty ideal, I take actions such as exercise, diet and more (Q25-2). I would like to be slimmer (Q25-3). < -I feel very comfortable with my body and I have no desire to change it (Q25-4).	0.689	1–5
Frequency of children’s food consumption	*** Vegetables (Q26-1). Fruit (Q26-2). Legumes, such as lentils, chickpeas, edamame, soy beans (Q26-3). Whole grains, such as wholegrain rice, whole wheat pasta, quinoa, wholegrain couscous (Q26-4). For school, pita/sandwich/roll, all whole grain (Q26-5). Nuts (Q26-6). *** < - Snacks (Q26-7). Sweets, such as confectionery, chocolate, cookies, ice-cream, cakes, sweet baked goods (Q26-7). Sweets, such as confectionery, chocolate, cookies, ice-cream, cakes, sweet baked goods (Q26-8). Processed meats (including vegetarian substitutes), such as sausages, hamburgers, salamis, schnitzel, ready to eat meatballs (Q26-9). Sweetened drinks, carbonated, concentrates, natural juices (Q26-10).	0.722	0–3

* Classification according to the degree of agreement (do not agree at all, 1; agree to a small extent, 2; agree to a certain extent, 3; agree to a large extent, 4; agree to a very large extent, 5. ** Classification according to true, 0/false, 1. *** Classification according to the frequency of consumption: almost every day, 3; several times a week, 2; a few times a month, 1; almost never or rarely, 0. < - Referring to the classification in the opposite direction. ^1^ The index is built from the two types of questions: classification according to the degree of agreement, and classification according to true/false; therefore, the degree of agreement has become binary for answers 1 and 2, versus 3 and above.

**Table 2:** Differences in the proportion of anthropometric categories among students studying in anthroposophical schools compared to conventional education schools.

BMI Percentile Categories	Total		Pv	Boys		Pv	Girls		Pv
	Conventional (*n* = 205,503)	Anthroposophical (*n* = 2247)		Conventional (*n* = 104,731)	Anthroposophical (*n* = 1111)		Conventional (*n* = 100,772)	Anthroposophical (*n* = 1136)	
Severe obesity	4.9	2.8	<0.001	5.5	3.2	0.001	4.4	2.4	0.001
Obesity	2.9	2.0	0.007	2.6	1.8	0.091	3.2	2.1	0.036
Overweight	11.2	9.6	0.014	10.6	9.7	0.348	11.9	9.4	0.012
Normal weight	79.1	82.9	<0.001	79.1	82.0	0.022	79.1	83.7	<0.001
Underweight	1.8	2.8	<0.001	2.2	3.2	0.008	1.4	2.4	0.005

BMI percentile categories were based on the World Health Organization (WHO) BMI growth charts as: severe obesity, BMI > 99th percentile; obesity, 97th < BMI ≤ 99th percentile; overweight, 97th ≤ BMI < 85th percentile; normal weight, 3rd < BMI ≤ 85th percentile; and underweight, BMI ≤ 3rd percentile.

**Table 3:** Demographic characteristics of parents of children in conventional vs. anthroposophical schools.

	Parents of Children in Anthroposophical Schools (*n* = 72)	Parents of Children in Conventional Schools (*n* = 142)	*p* Value
Female gender *n* (%)	64 (88.9%)	139 (97.9%)	0.005
Age (mean ± SD)	43.6 ± 4.7	41.4 ± 4.8	0.002
Number of children in the family (mean ± SD)	2.6 ± 0.7	2.8 ± 0.9	0.057
Parent’s education level *n* (%)			0.759
First degree	22 (30.5%)	42 (29.6%)	
Second degree	33 (45.8%)	66 (46.5%)	
Third degree	6 (8.3%)	14 (9.8%)	
Non-single parents *n* (%)	68 (94.4%)	134 (94.3%)	0.811
Family income *n* (%)			0.912
Much higher than average	17 (23.6%)	31 (21.8%)	
Higher than average	42 (58.3%)	80 (56.3%)	
Similar to the average	10 (13.9%)	21 (14.8%)	
Lower than average	2 (2.8%)	5 (3.5%)	
Much lower than average	1 (1.4%)	5 (3.5%)	
At least one of the parents is vegetarian or vegan *n* (%)	40 (55.6%)	44 (31.0%)	0.001

**Table 4:** Parental perception of their child’s dietary intake and food environment, and their own body image, among parents of children in conventional vs. anthroposophical schools.

Index	Parents of a Child in Anthroposophical Education (*n* = 72)		Parents of a Child in Conventional Education (*n* = 142)		*p* Value
	Mean ± SD	95% Confidence Interval	Mean ± SD	95% Confidence Interval	
Perceived role of the educational system in imparting a healthy lifestyle	4.09 ± 0.60	3.95–4.23	2.51 ± 0.78	2.38–2.64	<0.0001
Perceived health-promoting behaviors of teachers and the school curriculum	0.87± 0.11	0.84–0.90	0.28 ± 0.15	0.26–0.31	<0.0001
Perceived after-school birthday celebrations	0.86 ± 0.24	0.81–0.92	0.13 ± 0.20	0.10–0.17	<0.0001
Perceived after-school playdate-hosting norms	4.13 ± 0.68	3.97–4.28	2.51 ± 0.85	2.37–2.66	<0.0001
Perceived health-promoting behaviors in kindergarten	4.48 ± 0.76	4.30–4.66	3.20 ± 0.96	3.04–3.36	<0.0001
Perceived difference between the accepted eating pattern at home, and that of friends’ and family members	3.68 ± 0.93	3.33–3.88	3.66 ± 1.00	1.85–2.19	0.8629
Perceived parents’ body image	3.42 ± 0.86	3.86–4.34	2.90 ± 0.86	4.21–4.47	<0.0001
Frequency of children’s food consumption	2.35 ± 0.40	3.22–3.62	1.89 ± 0.42	2.75–3.04	<0.0001

**Table 5:** Main themes expressed by parents, according to their children’s educational system.

Quotes	Number of Responses (%)	Main Themes
Anthroposophical educational system (*n* = 65)		
Positive aspects of the educational system		
“The meals are rich and fresh with lots of color and variety. The kindergarten had a pleasant smell of cooking. The instilling of habits at a young age that there is always a tray of cut vegetables”	50 (76%)	Superior quality of food
“The children bake bread by themselves’ they see the full process of growing wheat: harvesting, grinding of stalks and transformation into flour. Then the children themselves prepare dough from the flour, they knead it, plait it, and put it into the oven and then eat what they themselves have made, fresh and warm. To see this complete process is an experience of connection to nature, creation, to myself as an entity as part of creation. An experience that the world is beautiful and good” “Involvement of the children in food preparation and setting of the table” “The fact that the child is a partner in meal preparation. Work in the garden, and placing in the compost”	29 (44%)	Involvement of the children in all food-preparation stages
“The rhythm of a fixed menu each day, the smell of food all day, the communal eating at the table”	28 (43%)	The surroundings promote healthy eating
Negative aspects of the educational system		
“There were no additional choices or variety for children who did not relate to the food offered” “The menu was very regulated, and sometime overly rigid, on the days that there was food that my son did not like, I knew that I would have to supplement with a lunch meal at home.”	14 (21.5%)	Fixed menu; no other choices
“Too many bread rolls” “Too many carbohydrates, the lactose is not necessary and too much bread”	7 (10.7%)	In some of the meals, there are a lot of carbohydrates
“There is butter at breakfast as this is an anthroposophical tradition A lot of dairy products” “There is no animal protein, no eggs, the lunch meal is sometimes too poor” “The food at the preschool is vegetarian, my children don’t particularly like legumes and soups, and so mainly eat the empty carbohydrates (rice, pasta, couscous without the soup), if there was a choice of meat or fish, their diet would be more balanced”	10 (15.2%)	Dispute regarding the amount of food of animal origin
Conventional education system (*n* = 135)		
Positive aspects of the educational system		
“The fact that there is food in the morning is amazing. Communal eating results in the child trying something he would not normally eat, and along with that, lack of success in causing my child to eat any fruit or vegetables” “A communal meal- the parents’ committee buys food products, and the teacher and aide together with the children, prepare the food. All children have the same food. Preparation of salad/soup at kindergarten”	45 (33.3%)	The morning meal is communal
“At lunch they would serve cut up vegetables that they gave to the children” “At the kindergarten, each child brings a fruit in the morning, and at 11:00 the teacher cut up the fruit and passed a tray with cut up fruit among the children”	33 (24.4%)	A lot of exposure to fruit and vegetables during the day
“There was a garden at the kindergarten, and the children planted vegetables there and later prepared soup and salads etc from them” “There was a vegetable garden where the children took part in the growing and tending”	21 (15.5%)	There is activity in the kindergarten’s vegetable garden
“There were clear instructions from the teacher not to bring sweets or sandwiches with chocolate, to kindergarten” “In the kindergarten, there was a ban on bringing sweets and snacks for the 10:00 in-between snack, and also not a sandwich with chocolate”	21 (15.5%)	The teaching staff limit the bringing of chocolate and sweets
“At the birthday parties there was only a cake, and no junk” “There was a ban on snacks, a ban on sweets at birthday parties, we were asked to bring fruit/vegetables (and a cake). At events at the kindergarten, there were no snacks, most of the parents were conscious (of this) and kept in line”	19 (14%)	Birthday parties without sweets and snacks (only a cake).
“Learning about the Food Pyramid and wise choices” “They spoke to them about a healthy lifestyle, and the importance of various foods”	14 (10.3%)	The children at the kindergarten learned about healthy nutrition
“There was a menu decided by the dietitian, and it was important to the staff there to offer the children a rich menu, healthy and varied” “A nutritionist accompanied the teachers and the aides”	11 (8%)	The menu was set by a dietitian and the menu was varied
Negative aspects of the educational system		
“At each celebration, there was a large number of snacks and cakes” “There was not always attention to the ban on bringing to the kindergarten, sweets and sweet foods, such as biscuits and chocolate” “Giving of sweet and sweet foods as reward for good behavior”	52 (38.5%)	Giving out of sweets and snacks at birthday parties and events, and particularly for good behavior
“As opposed to breakfast, lunch included portions of ultra-processed food. There was no control over the raw products, in comparison to breakfast” “In the afternoon program, the food was very processed and not tasty” “The food in the afternoon program was unacceptable, fatty and processed. After that, there were sandwiches with white bread and chocolate”	50 (37%)	Processed food was served

## Data Availability

The data presented in this study are available on request from the corresponding authors.

## Appendix A. Survey Questionnaire

Q1. I agree to take part in the study, and to answer the questionnaire.
  1. Yes
  2. No
Q2. Which city/town do you live in now? ___________________________
Q3. How many children do you have? ______________
Q4. How many of your children are currently studying in elementary school? _____
Q5. Are all the elementary schools your children are studying in located in the city/town where you live?
  1. Yes
  2. No
Q6. In which town/city is your children’s educational framework located? ___________
Q7. Please mark the elementary schools where your children study; (If some of the children are in different types of schools, you can mark more than one answer).
  1. State school
  2. State religious school
  3. Anthroposophy school
  4. Democratic school
  5. Other
Q8. Did one of your children study in the past, or is currently, in the Anthroposophy school?
  1. Yes
  2. No
Q9. Are all your children studying in the Anthroposophy school?
  1. Yes
  2. No
Q10. How many years ago did one of your children start studying at the Anthroposophy school? _______________
  Attention: Parents who have at least one child belonging to /studying at the Anthroposophy school are asked to answer questions, as related to the Anthroposophy school. If there is more than one child studying/ belonging there, ask them to relate to one child only.
Q11. In order to study at the school in the morning hours (not afternoon hours), is payment required?
  1. Yes
  2. No
Q12. Please indicate to what extent you agree with each of the statements, as related to the educational environment. 1 = Don’t agree at all, whereas 5 = Agree to a large extent, or “not relevant”.

	**1. Dont Agree at All (1)**	**2. Agree to a Small Extent (2)**	**3. Agree to a Certain Extent (3)**	**4. Agree to a Large Extent (4)**	**5. Agree to a Very Large Extent (5)**	**6. Not Relevant (6)**
1. I believe that the educational system imparts nutritional habits that outline a way of life						
2. I believe that teachers and care-givers in an educational setting should provide children with a healthy environment						
3. In my opinion, the teachers of my child’s school promote a healthy dietary pattern, which they maintain themselves						
4. In my opinion, the teachers of my child’s school care about his/her nutrition						

Q13. Please indicate tor each sentence if correct or incorrect or don’t know/not relevant The following questions relate to the role of the class convenor and the education system. Please indicate for each sentence if correct or incorrect or don’t know/not relevant.

		**Correct**	**Incorrect**	**Don’t Know/Not Relevant**
1	The teacher usually goes out with the class for a nature walk once a week			
2	Most mornings, students preform physical activity, such as jumping rope/running as part of the curriculum.			
3	Usually, the teacher takes the class to the garden, to plant fruit or vegetables. When it is time to harvest them, the children can taste them			
4	The school has strict guidelines regarding the nutritional content of food brought from home, and parents are instructed by the teacher about the permitted content of the lunch box/ between meal snack, birthday parties, festivals, trips.			
5	If nutrition guidelines are given to students and parents, the teachers usually monitor the implementation of the guidelines.			
6	In my children’s curriculum, there are lessons dedicated to lifestyle, nutrition and health.			
7	At school the concept of a healthy diet is integrated naturally into different activities, such as food preparation, songs, vegetable gardens, arts and crafts.			
8	The teacher sits and eats breakfast with the children.			
9	Before breakfast, the teacher gathers the children and carries out a small ritual/says a prayer.			
10	Sometimes, during meals at school, children watch a program or movie.			

Q14. The following questions relate to birthday celebrations. For each sentence, please indicate whether correct or incorrect, or don’t know/not relevant.

		**Correct**	**Incorrect**	**Don’t Know/Not Relevant**
1	Children which are born on the same month usually celebrate birthday parties together.			
2	When class birthday parties are held in the afternoon, the parents usually make sure to prepare homemade food, such as casseroles/soup, sandwiches, salads/vegetables, pasta etc. Later, there is also a birthday cake.			
3	When class birthday parties are held in the afternoon, snacks, sweets, vegetables, and sometimes pizza or sandwiches are served. Later, there is also a birthday cake.			

Q15. Please indicate to what extent you agree/disagree with each if the statements m as related to the school culture. 1 = don’t agree to 5 = agree to a very large extent or “not relevant”.

		**1. Dont Agree at All (1)**	**2. Agree to a Small Extent (2)**	**3. Agree to a Certain Extent (3)**	**4. Agree to a Large Extent (4)**	**5. Agree to a Very Large Extent (5)**	**6. Not Relevant (6)**
1	On school special ocations (such as a trip, graduation party, teachers’ day, birthday celebrations etc.) the food served usually includes snacks.						
2	Most parents who host kids for afternoon play-dates, offer fruit and vegetables.						
3	When my child is hosted for a play-date, snacks and sweets are often offered.						
4	In relation to other parents at the school, I am more careful regarding healthy nutrition.						
5	On school special occasions organizers try, as much as possible, to use reusable utensils.						

Q16. Please indicate to what extent you agree/disagree with each if the statements as related to the preschool of one of your children. 1 = don’t agree to 5 = agree to a very large extent or “not relevant”.

		**1. Dont Agree at All (1)**	**2. Agree to a Small Extent (2)**	**3. Agree to a Certain Extent (3)**	**4. Agree to a Large Extent (4)**	**5. Agree to a Very Large Extent (5)**	**6. Not Relevant (6)**
1	In preschool, children are encouraged to prepare different foods by themselves, such as cutting up vegetables/soup/picking olives/pastry/jam and others.						
2	At preschool, the children participate in all processes of food preparation including cleaning up.						
3	The food my child ate during his time at preschool was very healthy.						
4	My child’s teachers usually promote healthy nutrition, and they themselves have healthy dietary habits.						
5	My child’s teachers care about his/her nutrition.						
6	I feel that the teacher intervened too much in my child’s nutrition.						
7	I feel that the school continues with the same nutrition principles that were in the preschool.						

Q17. Write in a few words what, in the preschool, contributed to your child’s dietary habits and from the nutrition viewpoint, what were the positive aspects?
  _________________________________________________________________________________________________________________________________________________________________________________________________________________________________
Q18. Write in a few words, the negative aspects, from the nutrition viewpoint.
  _________________________________________________________________________________________________________________________________________________________________________________________________________________________________
Q19. Give a few examples of small things the education framework in the preschool did that were important, and changed the dietary habits of your child, or yours (as a parent). For instance, the food served at a birthday party, breakfast, lunch or anything else that comes to mind.
  ____________________________________________________________________________________________________________________________________________________________________________________________________________________________________________________________________________________________________________
Q20. Were there differences between the nutrition principles in the preschool and those in the school?
  ___________________________________________________________________________________________________________________________________________________________________________________________________________________________________________________________________________________________________________
Q21. Is there compatibility between the eating patterns and nutrition at home with that of the educational framework (school/preschool)?
  1. There is compatibility, it is in fact a direct continuation, and the principles are the same
  2. There is partial compatibility
  3. There is contradiction between what happens at home and what happens in school
Q22. Please mark the sentence which best describes your feelings in relation to the nutrition conduct in the educational framework (school/preschool).
  1. It frees me, gives me peace, and I feel good
  2. It doesn’t affect me/Not relevant
  3. I feel more pressured
Q23. Please indicate to what extent you agree with each of the following statements in relation to the interaction with other families, where 1 = don’t agree at all to 5 = agree to a very large extent or “not relevant”.

		**1. Dont Agree at All (1)**	**2. Agree to a Small Extent (2)**	**3. Agree to a Certain Extent (3)**	**4. Agree to a Large Extent (4)**	**5. Agree to a Very Large Extent (5)**	**6. Not Relevant (6)**
1	When my child is on a playdate at a friend’s or family member’s home, where the food served is different compared to that in my home, I feel uncomfortable.						
2	My child enjoys eating at other people’s homes when he is exposed to foods less heathy than the food served at home.						
3	I think my child feels conflicted when he meets with friends or family, where the food served is different than that served at home.						
4	I feel the need to adapt my point of view about nutrition, when I am in the company of other parents from school.						
5	I feel that other parents from school support my point of view about nutrition.						
6	I feel uncomfortable when I feel I should give my child snacks due to the company of parents from school.						

Q24. If you have something to add, we would be happy if you would specify: ___________________________________________________________________________
Q25. Please indicate to what extent you agree with each of the following statements in relation to body image, where 1 = don’t agree at all to 5 = agree to a very large extent or “not relevant”.

		**1. Dont Agree at All (1)**	**2. Agree to a Small Extent (2)**	**3. Agree to a Certain Extent (3)**	**4. Agree to a Large Extent (4)**	**5. Agree to a Very Large Extent (5)**	**6. Not Relevant (6)**
1	For me, it is important to be very slim, in accordance with the beauty ideal.						
2	In order to achieve the beauty ideal, I take actions such as exercise, diet and more.						
3	I would like to be slimmer.						
4	I feel very comfortable with my body and I have no desire to change it.						
5	It is very important to me that my children will be skinny, but I also don’t want to cause them to have eating disorders.						
6	If one of my children is or will be overweight, I prefer to go to a professional (dietitian/psychologist, etc.) so as not to cause them to have eating disorders.						
7	The educational framework relates to nutrition but less attention is paid to body image.						

Q26. With what frequency do the children eat the following foods at home?

		**Almost Daily**	**A Few Times/Week**	**Few Times /Month**	**Almost Never, or Very Rarely, Eat**
1	Vegetables				
2	Fruit				
3	Legumes, such as lentils, chickpeas, edamame, soy beans				
4	Whole grains, such as wholegrain rice, whole wheat pasta, quinoa, wholegrain couscous				
5	For school, pita/sandwich/roll, all whole grain				
6	Nuts				
7	Snacks, such as Bissli, Bamba, pretzels				
8	Sweets, such as confectionery, chocolate, cookies, ice-cream, cakes, sweet baked goods				
9	Processed meats (including vegetarian substitutes), such as sausages, hamburgers, salamis, schnitzel, ready to eat meatballs				
10	Sweetened drinks, carbonated, concentrates, natural juices				

Q27. Please mark if one of the parents is
  1. Vegetarian
  2. Vegan
  3. Not vegetarian, but reduces consumption of food of animal origin
  4. Eats a variety of food, of both plant and animal origin
  5. Other
Q28. How would you define your family’s dietary pattern?
  1. Very healthy
  2. Healthy
  3. Regular
  4. Not so healthy
  5. Not healthy
Q29. How important is it to you that your children should eat in a healthy way?
  1. Very important
  2. Important
  3. Important to a certain extent
  4. Important to a minor extent
  5. Not important
Q30. How important is it to you that in the education framework there be an emphasis on healthy food?
  1. Very important
  2. Important
  3. Important to a certain extent
  4. Important to a minor extent
  5. Not important
Q31. Was your choice of a particular type of education related to the nutrition aspect?
  1. Yes
  2. No
  3. Not relevant
Q32. Do you want to change the dietary habits of your immediate family or the extended family?
  1. No
  2. Yes, specify:______________________________________________________
Q33. Sex
  1. Male
  2. Female
  3. Other
Q34. Are you
  1. Jewish
  2. Arab
  3. Druze
  4. Bedouin
  5. Other, specify:__________________________________________________
Q35. How would you describe your state of religiosity?
  1. Secular
  2. Traditional
  3. Religious/national religious
  4. Ultraorthodox
  5. Other
Q36. What is your level of education?
  1. Elementary or less
  2. Partial high school
  3. Completed school, without matriculation
  4. Competed high school, with matriculation
  5. Post school- teachers college, nursing school, technical school for engineers
  6. First Degree
  7. Second degree
  8. Doctorate
Q37. What is your age? ____________________
Q38. What is your personal status?
  1. Married
  2. Living with a partner
  3. Divorced or separated
  4. Single
  5. Widowed
Q39. What is the age of your spouse/partner? __________________
Q40. In comparison to a gross family income of 14700 shekels a month, your income is
  1. Our family income is much lower than this amount
  2. Our family income is lower than this amount
  3. Our family income is about that amount
  4. Our family income is higher than that amount
  5. Our family income is much higher than that amount
Q41. Are you or your spouse in a nutrition-related profession?
  1. Yes, specify:_______________________________________________
  2. No
Q42. Are you or your spouse involved in one of the therapeutic professions, such a social worker/psychologist/art therapist, etc.?
  1. Yes, specify;_____________________________________________________
  2. No
